# A Randomized Double Blinded Placebo-Controlled Clinical Trial of a Probiotic or Metronidazole for Acute Canine Diarrhea

**DOI:** 10.3389/fvets.2019.00163

**Published:** 2019-06-04

**Authors:** Justin Shmalberg, Christina Montalbano, Giada Morelli, Gareth J. Buckley

**Affiliations:** ^1^Department of Comparative, Diagnostic, and Population Medicine, College of Veterinary Medicine, University of Florida, Gainesville, FL, United States; ^2^Department of Animal Medicine, Production and Health, University of Padua, Padova, Italy; ^3^Department of Small Animal Clinical Sciences, College of Veterinary Medicine, University of Florida, Gainesville, FL, United States

**Keywords:** probiotic, diarrhea, metronidazole, colitis, canine (dog), dietary indiscretion, acute diarrhea, randomized clinical trial (RCT)

## Abstract

Acute diarrhea is a common, often self-limiting, cause of presentation for veterinary care, yet there is a paucity of data on frequently-prescribed treatments. The purpose of this randomized, double blinded placebo-controlled clinical trial was to compare two anecdotally-recommended treatments: a probiotic combination and metronidazole. Sixty dogs without concurrent comorbidities were randomized into three treatment groups. The time to resolution of diarrheal signs was evaluated using owner surveys and fecal scoring charts. Dogs presenting with acute diarrhea achieved acceptable fecal consistency after 3.5 ± 2.2 days when receiving probiotic, 4.6 ± 2.4 days with oral metronidazole, and 4.8 ± 2.9 days with placebo; statistically significant differences were not identified between treatment groups (*p* = 0.17). These findings failed to provide evidence for the common use of metronidazole in this cohort of dogs with acute canine diarrhea, and a larger study population would be required to identify a statistically significant effect of probiotics.

## Introduction

Much of the treatment of acute canine diarrhea remains empiric and confounded by the self-limiting nature of the condition (1–3). Helminthic or protozoal infections and primary bacterial or viral enteritis may cause acute diarrhea in dogs, but in the majority of cases a cause is inapparent (2–4). The relative risk for diarrhea may be influenced strongly by age, breed, gender, environment, and season (5–7). Dietary indiscretion or housing stress (such as a kennel or shelter environment) are also predisposing factors (8). Acute diarrhea and potentially its resolution may be affected by either primary or secondary changes in the fecal microbiota, an area presently receiving substantial research interest (9–11). Both antimicrobial drugs and probiotics are administered frequently in the treatment of acute diarrhea, with the goal of influencing the gastrointestinal microbiome and shortening the time to clinical resolution (2, 12).

Probiotics may represent a viable alternative treatment strategy to antibiotic administration given concerns about antimicrobial efficacy and resistance in acute diarrhea (3, 12). Probiotics are classically defined as live, viable microorganisms that produce evidence of a health benefit when administered (13). Variable effects in dogs have been observed for the strains from the following bacterial genera: *Lactobacillus, Enterococcus, Bacillus, Streptococcus, Bifidobacterium, Pediococcus*, and *Weissella* (14–16). Clinical studies evaluating the impact of probiotics suggest possible prophylactic effects on diarrhea resulting from intense exercise or shelter environments (12, 17). A recent placebo-controlled study concluded that probiotics may reduce the duration of clinical signs in dogs administered a combination probiotic for treatment of acute diarrhea from multiple causes (18). In a similar study, improved fecal quality following administration of a probiotic fermented milk product was reported (19). However, probiotic effects are likely condition- and strain-specific (12).

Metronidazole is a nitroimidazole antibiotic empirically prescribed for the treatment of acute and chronic canine diarrheal diseases (20–24). The mechanism of action for both antibacterial and antiprotozoal effects remains incompletely understood, but it is believed to inhibit bacterial DNA and induce oxidative damage primarily in anaerobic bacteria (25). The drug's effect on both *Giardia* and *Clostridium* spp. make it a theoretically attractive choice as both have been suggested as causative diarrheal pathogens (4, 26–29). Metronidazole may also have immunomodulatory or anti-inflammatory effects but no difference was detected in dogs with inflammatory bowel disease given prednisone and metronidazole vs. prednisone alone (21, 24). Administration to healthy dogs causes significant changes in the microbiome, some of which persist following discontinuation of the drug with unknown clinical effects (29, 30). A single prospective randomized blinded trial in dogs with acute hemorrhagic diarrhea failed to demonstrate a beneficial effect with metronidazole administration in addition to amoxicillin-clavulanic acid and supportive therapy (31). The use of metronidazole in acute non-hemorrhagic canine diarrhea has not been subjected to a placebo-controlled trial.

The purpose of this study was to evaluate a combination probiotic with a greater number of strains and higher number of colony forming units (CFU) than those used in similar studies for the resolution of acute uncomplicated diarrhea in dogs as compared to an alternate treatment, metronidazole, and to placebo.

## Materials and Methods

Client-owned dogs presenting to either of two emergency centers of a university veterinary academic teaching hospital were recruited for enrollment in a randomized, double-blinded, placebo-controlled clinical trial between March 2017 and August 2018. Eligibility criteria evaluated by the attending clinician included acute signs (< 7 days) of diarrhea with or without concurrent vomiting, a body weight of 4–45 kg, and the lack of clinically-relevant comorbidities which would have been expected to cause secondary diarrhea (endocrinopathies, organ dysfunction, immune-mediated disease, suspected pancreatitis based on severe abdominal pain, etc.). Dogs with clinical signs consistent with severe acute hemorrhagic diarrhea syndrome characterized by significant dehydration, hypovolemia, or subjectively large volumes of hematochezia were excluded. Eligibility was at the discretion of the attending clinician at the time of presentation, and owners were informed of the study design and consented to participation in the study which was approved by a hospital research review committee and an Institutional Animal Care and Use Committee (IACUC).

Clients were provided a financial incentive for participation, and agreed to answer a survey at presentation and to participate in phone interviews at 5 and 10 days after initiation of treatment. Owners were provided a fecal scoring chart and asked to observe all defecation events and to record the day when the feces was first consistent with a score of 3 or less on the provided chart, which represents feces that has form (32, 33). Owners were not asked to record fecal scoring daily nor to record frequency of defecation as the study endpoint was formed feces. Survey questions included the duration of diarrheal signs, any previous history of diarrhea, possible triggering events (dietary indiscretion, intentional diet change, stress), and owners were asked to indicate any clinical signs which included hematochezia, melena, tenesmus, change in defecation frequency, the presence of mucus, and the subjective water content of the feces (watery or semi-formed). Additional data collected on each dog included age, sex, breed, spay or neuter status, and body weight.

Blood was collected on enrolled dogs for a complete blood count and a serum biochemistry. Feces were obtained when possible at the time of examination for a fecal flotation and a commercial PCR gastrointestinal panel (Canine Diarrhea RealPCR Panel, IDEXX Laboratories, Westbrook, ME) for detection of *Campylobacter coli, Campylobacter jejuni*, canine circovirus, canine distemper virus (CDV), canine enteric coronavirus (CECoV), canine parvovirus 2 (CPV-2), *Clostridium difficile* toxin A/B, *Clostridium perfringens* alpha toxin (CPA), *Clostridium perfringens* enterotoxin (CPE), *Clostridium perfringens* netEF gene (CPnetEF), *Cryptosporidium* spp., *Giardia* spp., and *Salmonella* spp. Clinicians were permitted, at their discretion, to administer fluid therapy (subcutaneous or intravenous), fenbendazole, and/or maropitant in order to maximize clinician and client enrollment; all treatments and examination findings were recorded in the medical record. Dogs were withdrawn from the study due to owner non-compliance with treatment administration, abnormalities noted on post-enrollment laboratory or PCR testing which supported a diagnosis other than uncomplicated diarrhea, or the presence of more than occasional parasite ova in a fecal sample. Dogs were enrolled until 20 were present in each group, based on the results of a pre-study power analysis of the group size likely to produce a clinically significant reduction of 2 days in time to normal feces using the mean and standard deviation from previous studies (5.6 vs. 6.6 days, SD = 2.3 days, alpha = 0.05, beta = 0.2) (22). The study was unblinded to the investigators after the enrollment of 60 dogs to perform statistical analyses.

Dogs were assigned, using a pre-study randomization schedule obtained from a random sorting feature of a commercial software program (Excel for Mac, version 15.25, Microsoft, Redmond, WA), to receive one of three treatments which included a commercial probiotic product, oral metronidazole, or placebo; both attending clinicians, the clinician obtaining follow-up, and the owners were blinded. All treatments were administered twice daily for 10 days with the first dose administered following the initial examination, irrespective of whether the patient was immediately discharged to owner care or hospitalized. Probiotic (Vital Vet, Vital Planet, Palm Harbor, FL) was provided in vegetable-based capsules with a label guarantee of 30 billion colony forming units (CFU), and a measured value of 70 billion CFU at initiation of study, per capsule of a mixture of the following bacterial strains: *Bifidobacterium bifidum* VPBB-6, *Bifidobacterium longum* VPBL-5, *Bifidobacterium animalis* VPBA-4, *Bifidobacterium infantis* VPBI-6, *Lactobacillus acidophilus* VPLA-4, *Lactobacillus plantarum* VPLP-5, *Lactobacillus casei* VPLC-1, *Lactobacillus brevis* VPLB-5, *Lactobacillus reuteri* VPLR-1, *Lactobacillus bulgaricus* VPLB-7. Probiotic capsules contained 425 mg of a blend of organic acacia gum and fructo-oligosaccharides (FOS). Metronidazole powder was given in gelatin capsules according to a weight-range administration schedule; 125 mg was administered twice daily to dogs between 4 and 10 kg, 250 mg to dogs between 10.1 and 20 kg, and 400 mg for dogs 20.1–45 kg. Placebo capsules contained sucrose at an equal volume to probiotic and metronidazole capsules. Capsules were not visually different and were prepared by licensed pharmacists (metronidazole) or the probiotic manufacturer (probiotic and placebo). All dispensed vials were labeled with instructions and with a letter group assignment as the only identifying treatment indication.

Owners were asked to monitor their dogs daily following discharge for fecal quality, and to report any worsening to the emergency service. Dogs were hospitalized based only on owner preference or to facilitate clinician-ordered treatments, and the days of hospitalization were recorded. Owners were provided standardized discharge instructions of feeding instructions which suggested a 24 h fast followed by slow re-introduction of food over the subsequent 24 h. If diarrhea significantly worsened based on the owners' report of increased frequency, worsened fecal scoring, increased hematochezia, or worsened straining, owners were given the option of rescue antibiotic treatment during the 10 day treatment period or at study completion (day 10) if diarrhea had not resolved (tylosin, 30 mg/kg PO twice daily for 10 days). Clients were asked to return to the hospital if the patient's condition deteriorated, and they were informed that there would be no charge for follow-up medications or treatments. Examples of deterioration provided to owners included lethargy, persistent vomiting, melena, inappetance of >48 h duration, and any other concerning behaviors or symptoms. A survey was sent 6 months after enrollment of the last dog to determine if another diarrhea episode occurred following study completion.

Probiotic potency was tested by the manufacturer at 8 and 18 months to evaluate the effects of storage time given that all probiotics were prepared at the start of the study period. Such testing was performed without knowledge of the study authors, using probiotic viability testing at an outside laboratory.

### Statistical Analysis

Data were assessed for normality with an Anderson-Darling analysis. Parametric group data were compared with one-way analysis of variance (ANOVA) and non-parametric data with Kruskal-Wallis; statistical significance was established if the probability of error was ≤ 0.05. Parametric data was reported as the mean and standard deviation. Two-proportion *z*-tests were performed between each treatment group for the following observations: a previous history of diarrhea, inciting cause (dietary indiscretion, stress, or food change), vomiting, hematochezia, fluid therapy, maropitant administration, fenbendazole administration, submission of fecal flotation and fecal PCR, the presence of *Toxocara canis*, CPA, CPE, CPnetEF, *Campylobacter coli*, and circovirus with significance established using Bonferroni correction (*p* < 0.017). Freeman-Halton-Fisher exact probability testing was performed to assess any difference in the number of dogs in each treatment group with diarrhea in the 6 month post-study period with a *p* < 0.05 considered significant (34). In order to evaluate the effects of treatments as well as possible confounding variables associated with the time required to achieve formed feces, a backwards linear regression analysis was performed. The following factors were considered in addition to treatment group: age, body weight, previous history of diarrhea, the type of inciting cause (dietary indiscretion, stress, intentional food change), vomiting, hematochezia, duration of clinical signs, fluid therapy, anti-emetic administration, parasite ova, and the presence of *C. perfringens* toxins. Dichotomous variables were dummy coded for entry into the regression model. Variables were removed from the model if their significance was *p* > 0.2. Variables were included in the final model if the *p*-value at each iteration was below 0.1. All analyses were performed using commercially-available statistical software (Minitab Express v.1.5.1, Minitab, State College, PA).

## Results

A total of 63 dogs were enrolled in the study and 60 dogs successfully completed the study, divided equally among all three treatment groups. Three dogs were withdrawn and not included in the final analysis due to either a significant parasite burden (*n* = 1, metronidazole group) or failure of owners to give the assigned study treatment (*n* = 2, one each in probiotic and placebo group). Group comparisons are summarized in Table 1, and no differences were detected between groups for any hematological or biochemical analyte (*p* = 0.12–0.74). Dogs presenting with acute diarrhea achieved acceptable fecal consistency after 3.5 ± 2.2 days when receiving probiotic, 4.6 ± 2.4 days with metronidazole, and 4.8 ± 2.9 days with placebo (*p* = 0.17). The mean metronidazole dose was 17.8 ± 4.6 mg/kg (range: 11.2–24.0 mg/kg) twice daily. All dogs were treated on an outpatient basis with no hospitalization time >12 h. No adverse effects in any treatment group were noted, and no dogs required rescue treatment with tylosin.

**Table 1:** Comparison of patient characteristics, history, additional treatments, laboratory testing, and time to formed feces between study treatment groups.

	**Probiotic (*n* = 20)**	**Metronidazole (*n* = 20)**	**Placebo (*n* = 20)**	***p*-value^††^**	**All Treatment Groups (*n* = 60)**
Time to formed feces (days)	3.5 ± 2.2	4.6 ± 2.4	4.8 ± 2.9	0.17	
Age (yrs)	5.3 ± 3.2	5.7 ± 3.7	5.7 ± 3.9	0.94	5.6 ± 3.6
Body weight (kg)	20.2 ± 11.3	22.8 ± 13.0	20.9 ± 11.6	0.78	21.2 ± 11.8
Duration before presentation (days)	1.7 ± 1.4	1.8 ± 2.3	1.6 ± 1.4	0.94	1.7 ± 1.4
Previous history of diarrhea (*n*)	10	11	13	ns	34
Dietary indiscretion (*n*)	9	4	8	ns	21
Stress (*n*)	3	4	2	ns	9
Food change (n)	1	3	5	ns	9
Vomiting (*n*)	10	10	8	ns	28
Hematochezia (*n*)	14	13	12	ns	39
Fluid therapy (*n*)	8	6	9	ns	23
Maropitant (*n*)	11	11	8	ns	30
Fenbendazole (*n*)	1	2	3	ns	6
Hematocrit (%) (ref: 37.3–61.7)	50.2 ± 5.4	49.7 ± 6.9	47.4 ± 8.3	0.42	49.1 ± 7.0
Total protein (g/dL) (ref: 5.2–8.2)	6.7 ± 0.5	6.8 ± 0.7	6.6 ± 0.6	0.71	6.7 ± 0.6
Creatinine (mg/dL) (ref: 0.5–1.8)	1.0 ± 0.3	1.1 ± 0.3	1.1 ± 0.3	0.25	1.1 ± 0.3
Sodium (mEq/L) (ref: 142–160)	149 ± 3.9	151 ± 2.9	150 ± 2.5	0.14	150 ± 3.2
Potassium (mEq/L) (ref: 3.5–5.8)	4.1 ± 0.6	4.5 ± 0.7	4.3 ± 0.5	0.12	4.3 ± 0.6
Chloride (mEq/L) (ref: 107–122)	114 ± 3.7	115 ± 3.4	115 ± 3.5	0.59	115 ± 3.5
*Toxocara canis* ova^†^	0/17	2/15	2/17	ns	4/49
CPA positive^†^	8/9	10/11	13/15	ns	31/35
CPE positive^†^	3/9	5/11	8/15	ns	16/35
CPnetEF positive^†^	2/9	4/11	6/15	ns	12/35
*Campylobacter coli* positive^†^	0/9	0/11	1/15	ns	1/35
Circovirus positive^†^	0/9	0/11	2/15	ns	2/35
Episode of diarrhea after study (*n*)	4	1	3	0.48	8

† *numbers given are the n positive/n samples submitted. Not all dogs had sufficient sample for analysis*.

†† * No results were statistically significant; ns indicates there were no significant differences for pairwise two-proportion z-tests which were assessed using Bonferroni correction (p < 0.017 considered significant). CPA, C. perfringens alpha toxin gene; CPE, Clostridium perfringens enterotoxin (CPE); CPnetEF, Clostridium perfringens netEF gene (CPnetEF)*.

The linear regression identified a history of previous diarrhea episodes (β = 1.8, *p* = 0.05), dietary indiscretion (β = 2.2, *p* = 0.03), fluid therapy (β = −2.6, *p* = 0.01), and positive fecal flotation (β = 3.8, *p* = 0.02) as statistically significant categories to include in the final model. Treatment group did not significantly affect the duration of diarrhea after adjusting for confounding factors (*p* = 0.14). The linear regression process was also repeated following removal of all dogs with positive fecal flotations. A history of previous diarrhea (β = 1.6, *p* = 0.01), dietary indiscretion (β = 1.4, *p* = 0.01), and fluid therapy (β = −1.7, *p* = 0.04) remained in the model with no other new factors. Treatment group was not a significant factor after adjusting for confounding factors (*p* = 0.56). After removal of dogs with parasite ova, dogs in the probiotic group achieved normal fecal consistency after 3.5 ± 2.2 days, 4.5 ± 2.0 in the metronidazole group, and 4.8 ± 3.0 in the placebo group (*p* = 0.21).

*C. perfringens* alpha toxin gene (CPA), *C. perfringens* enterotoxin gene (CPE), and *C. perfringens* netEF gene (CPnetEF) were detected in a number of patients (Table 1), with a mean gene quantity of 4.9 × 10^6^, 4.2 × 10^6^, and 12.8 × 10^6^ copies with a maximum of 83 × 10^6^, 31 × 10^6^, and 93 × 10^6^, respectively. These values represent 57.1% (*n* = 20), 22.8% (*n* = 8), and 31.4% (*n* = 11), respectively, of sample tests above the cut-off suggested by the test provider for an association with diarrhea. All dogs positive for CPE or CPnetEF were also positive for CPA. No dogs were positive for *Campylobacter jejuni, Clostridium difficile* toxin A/B, canine distemper virus (CDV), canine enteric coronavirus (CECoV), canine parvovirus 2 (CPV-2), *Cryptosporidium* spp., *Giardia* spp., or *Salmonella* spp.

Forty percent of owners responded to requests for long-term follow-up (*n* = 24) following study completion. Diarrhea was reported in the follow-up period in 33.3% of dogs for which information was available (Table 1, *n* = 8).

There was substantial deterioration of the probiotic product over time which was not reported by the manufacturer to the study authors until study completion. There were 70 billion CFU per capsule when the study was initiated. Laboratory analysis by the manufacturer after 8 months of storage, at which time half of dogs were enrolled in the probiotic group, revealed a concentration of 6 billion CFU, and no detectable viable colonies after 18 months of storage. However, there was no significant difference in the time to formed feces between the first half (3.6 ± 2.9 days) and second half (3.3 ± 1.4 days) of dogs enrolled in the probiotic group (*p* = 0.84).

## Discussion

Neither the probiotic product nor metronidazole achieved a statistically significant improvement in the time to clinical resolution of the patients enrolled in this study. There was a large standard deviation in the response to treatments, which was consistent with two previous studies which evaluated probiotic vs. placebo in acute diarrhea (18, 22). Improvements previously seen with probiotics were not identified here, but the study was underpowered to detect a difference of one day.

The data suggest that although the treatment interventions did not produce a statistically-significant difference, other factors including a previous history of diarrhea, dietary indiscretion, fluid therapy, and parasite ova may have confounded the results. These findings should be interpreted cautiously given the small sample size. An effect from a previous history of diarrhea may suggest inherent susceptibility and possibly chronically altered microbiota. Over half of the enrolled dogs in the present study had a previous history of diarrhea, which is consistent with another investigation that reported a recurrence rate of 38.5% of acute diarrhea in a 1 year period (35). Dietary indiscretion, scavenging, and food changes are known risk factors for diarrhea but have not previously been shown to affect the duration of signs (8, 36). This may support different mechanisms for stress- or kennel-associated diarrhea as opposed to that related to dietary intake. A limitation of the present study is that dogs were not screened for pancreatitis using serum-based methods, however, dogs with clinical signs suggestive of pancreatitis were to be excluded so it is unlikely that dietary indiscretion influenced severe co-morbidities that were undetected (37). Additional study on whether fluid therapy truly impacts the duration of diarrhea would be necessary.

Gastrointestinal parasites may have affected time to resolution, even though dogs with significant ova burden were excluded from the study. This occurred despite treatment with fenbendazole. Previous studies suggest that endoparasites do not influence the occurrence of gastrointestinal signs (8, 21, 27). However, one study identified *Toxocara canis*, the parasite in the four positive study cases, only in diarrheal dogs and not in control dogs, and another study identified it in an uncontrolled cohort of diarrheal dogs (4, 38). The reason for the longer duration of diarrhea in dogs with parasites in the present study remains unclear but could be due to the parasites causing a more variable duration of diarrhea by impacting the gastrointestinal mucosa or microbiome. Conflicting data exist on the influence of helminths on the human microbiome and host immune function (39). Dogs with low parasite ova were included in this study to shorten the enrollment period and because of the questionable effect on producing clinical signs of acute diarrhea when present in adult dogs in low amounts. However, the number of detected ova may not have been commensurate with overall parasite burden. Although study results were unchanged when these dogs were removed from the data set, future studies of probiotics or metronidazole should exclude all dogs with positive parasite detection.

Significant pathogens were not identified with PCR testing. *Campylobacter coli*, identified in one dog, may have been incidental as normal dogs showed higher cultures of *Campylobacter* spp. compared to dogs with diarrhea (40). Canine circovirus was identified in two dogs, but does not appear independently correlated to severe hemorrhagic diarrhea (40, 41). No dogs were detected with *Giardia* spp. This is surprising given the prevalence described in other screening studies (4, 26–28, 38).

The presence of *Clostridium* spp. and associated toxins in all groups is expected although differences were detected in this population as compared to those in other studies. CPA and CPE are known to occur in both diarrheal populations and in healthy dogs (9, 10, 19, 21, 27, 28, 40, 42–46). Direct detection of *Clostridium perfringens* enterotoxin was identified in 45 vs. 25%, 28 vs. 5%, 11 vs. 10%, 16 vs. 1% of dogs with diarrhea vs. controls in four studies (4, 40, 42, 47). The detection of the CPA gene using PCR was higher (87%) in this study than in other reports; CPA gene was detected in 39 vs. 14% of controls in one study and in 75% of diarrheal cases in another (19, 28). CPE gene copies were detected in 46% of enrolled dogs, which is largely consistent with previous reports of 40–65% in dogs with diarrhea, including hemorrhagic diarrhea, and 39–40% in dogs without gastrointestinal signs (19, 27, 45). Four percent and 2% of healthy dogs have been reported to have a CPA and CPE above the cutoff, respectively, and causality between the presence of these genes and disease duration or severity has not been established (43, 46, 48). Thirty four percent of fecal samples tested in the present investigation had detectable CPnetEF genes. The netF encoding gene has been previously identified in 7.6% of dogs with diarrhea and in 57% of dogs with acute hemorrhagic diarrhea syndrome (49, 50). No *Clostridium difficile* was detected by PCR in this study. The prevalence is largely unknown with current estimates between 10 and 21% in diarrheal dogs (40, 46, 49).

The widespread use of metronidazole in simple uncomplicated diarrhea, or diarrhea in the absence of systemic signs or significant hemorrhagic diarrhea syndrome, with or without *C. perfringens* and its related toxins was not supported by the data in this small study cohort. No previous reports have compared metronidazole directly to placebo, and in the present study, 4.6 vs. 4.8 days would be clinically insignificant even if adequate numbers were to show statistical significance. The minimum dose used in all patients was consistent with empiric dosing for non-specific diarrhea (10–15 mg/kg twice daily), but lower than that employed by other studies of 20-25 mg/kg twice daily (2, 21, 23). No neurologic signs were appreciated, but higher doses (>60 mg/kg/d) have been associated with such side effects (51). Previous studies have used metronidazole as a control or optional treatment when evaluating probiotics (12, 21, 22). Metronidazole, in addition to the lack of proven benefits, has demonstrated mixed effects on the healthy canine microbiome (29, 52). Antibiotics, specifically amoxicillin and clavulanic acid, did not show favorable effects in more severe canine hemorrhagic diarrhea (53). This is in contrast to probiotics which accelerated a faster reduction of netF encoding *C. perfringens* although dogs without antibiotic treatment also resolved (50). The results of this study along with the other available data suggest metronidazole should not be used except in cases with a very specific indication, such as the treatment of *Giardia* spp., but other treatments may also be available with a more limited spectrum in those infections (26).

The mean time to resolution was 1.3 days shorter in dogs that received probiotics vs. placebo, but statistical significance was not established. If such a difference exists, which would be clinically significant, more than 60 dogs in each group would be required, and therefore this study was underpowered. The time to resolution in the present investigation was similar to another probiotic study of client-owned acute diarrheal dogs which reported 3.9 ± 2.3 days for resolution of diarrhea in patients receiving a probiotic. However, in this previous study, the placebo group took a significantly longer time to achieve clinical resolution of diarrhea (6.6 ± 2.7 days) compared to the current study (22). That cohort was comprised of younger dogs residing at a guide-dog training facility and may not be directly comparable to the dogs enrolled in the present trial. Another study demonstrated much shorter probiotic and placebo recovery periods (1.3 vs. 2.2 days) but this may be partially due to a longer duration of clinical signs before enrollment in that study (3.1 days) vs. in the current (1.7 days) (18). In addition, dogs in that study were excluded if the attending clinician identified the need for any other supporting treatments or for hospitalization. Studies on shelter dogs have been mixed, but are likely not comparable to client-owned animals given the different etiology and time of presentation (12, 21, 54). Clinical improvement was noted one day earlier in dogs administered a probiotic with higher CFU for more severe hemorrhagic diarrhea vs. placebo (50). Direct comparative interpretation is often difficult due to different strains and concentrations of probiotics (12).

The decline in probiotic CFUs may have been due to the lack of a desiccant packet and air-tight sealing which is typical of commercial products but which were not included in this study due to the standardized study-specific packaging (Watson, personal communication). Differences were not detected between the first and second half of dogs enrolled in the probiotic group, which may be explained by the variable response times within the group, the low power of this comparison, or the lack of efficacy of the product at any CFU potency. This information was unknown to the researchers until study completion, when provided by the probiotic manufacturer to the authors, and therefore corrective action was not possible during the study. The impact of probiotic concentration and viability is unclear with research studies in other species demonstrating benefits from dead bacteria cells (55). Therefore, the degraded product should likely not be considered equivalent to placebo. A mismatch between commercial probiotic claims and actual potency appears common in the pet supplement market, but in this case was likely due to the inadvertent lack of normal preservation techniques used in the commercial version of this product (56). Past studies have not reported potency throughout the study duration, and probiotics may have similarly degraded. In addition, optimal probiotic dosing has not been established. Previous studies of diarrhea have provided 2–12 billion CFU for an average-sized dog, which was less than the starting concentration in this study (18, 19). Future studies should monitor probiotic viability at established intervals during the study and replenish probiotic product when necessary to maintain potency, or precautionary measures should be taken to ensure prolonged viability over the label claim threshold.

The probiotic capsule contained potential prebiotics, or fermentable fibers, in the form of acacia gum and FOS which were not found in the placebo. This confounds the evaluation of probiotic strains given that fiber may have effects on the bacterial mass and composition of the microbiome (42). Acacia gum is a rapidly fermentable soluble fiber, but for the smallest dog in the study, this fiber would contribute no more than one percent on a dry matter basis of total estimated dietary intake and considerably less in a study dog of average size (estimated 0.4% dry matter) (57). Dietary studies in dogs have evaluated much larger amounts of gum than used in this probiotic product (58). Similarly, FOS has been shown to cause increased bacterial fermentation and bacterial shifts but published investigations varied in the inclusion rate of this fiber from 0.5 to 1.5% DM, higher than that estimated to have been administered in the present study (59–61). Future studies may better evaluate the independent impact of probiotic and prebiotic combinations by inclusion of similar prebiotics in the placebo.

The study design is subject to a number of other limitations. The total number of diarrhea cases presented to this tertiary referral center were not recorded during this period given the large case volume and the number of cases presenting with diarrhea secondary to an excluding concurrent condition (foreign body, toxicity, etc.). Similarly, the number of owners declining participation was not tracked given the large number of clinicians attending to such cases, and therefore selection bias could be present. Microbiome and metabolome testing were not performed before or after treatment which might elucidate the course of the disease and the impact on potentially pathogenic genera like *Clostridium*. The impact of previous episodes of diarrhea might also have been explained with such data. The fecal testing which was performed was only possible in some dogs because of insufficient sample; in retrospect, PCR swabs of the colon should have been obtained, and PCR for helminths could also have been performed. The clinician discretion to administer fluids, maropitant, or empiric fenbendazole were all potentially confounding variables that would have been eliminated with more stringent inclusion protocols. Additional dogs in each group would be necessary to achieve adequate power between probiotic and placebo, and additional numbers would potentially permit stratification by inciting cause to assess differences between stress- or dietary indiscretion-related diarrhea. The use of owner reports of fecal consistency is subject to potential imprecision and to bias; the use of dogs with induced colitis would provide more homogeneity but fail to reproduce the diversity of disease seen in a clinical setting. Case bias may also have occurred as all cases presented to a tertiary referral center and therefore could represent more refractory or severe cases than those which might be encountered in primary opinion practice. Owner observation was limited to whole treatment days and therefore could have occurred earlier or later in the day, and could be subject to recall bias. The collection of fecal scoring data on a daily basis was not performed, but would have provided additional data albeit at the expense of increased effort by owners and suspected reduced owner compliance as a result. Finally, diet was uncontrolled in the treatment period and information was not collected regarding the dietary interventions of the owner. Consequently, the amount of caloric, nutrient, and fiber intake could have influenced both the fecal microbiome as well as clinical outcome (42, 62, 63). Additional studies should control for diet when possible. The limitations described herein are consistent with the multifactorial nature of diarrhea and its treatment as well as the inherent difficulties in assessing dogs returned to their owners.

The use of metronidazole in acute diarrhea does not appear warranted in this cohort characterized by mild to moderate signs of hemorrhagic or non-hemorrhagic acute diarrhea, based on the results of this placebo-controlled trial. Its use should be discouraged until evidence-based data demonstrate a difference in treatment outcome for dogs with acute diarrhea. Similarly, no difference was detected in the probiotic group although the study was underpowered and a larger study should be performed. No adverse effects from any intervention were noted.

## Ethics Statement

The study was performed after the University of Florida institutional animal care and use committee (IACUC) approved the study following the guidelines for animal use according to the IACUC. Client owned dogs were enrolled after informed consent in accordance with the Declaration of Helsinki using a form approved by the University of Florida Veterinary Hospitals Research Review Committee.

## Author Contributions

JS was responsible for conception of the study, data interpretation, supervision of data collection, statistical analysis, and drafting the manuscript. CM and GM were responsible for data collection and analysis, enrollee communication, review of relevant literature, and manuscript revision. GB was responsible for recruitment of patients and manuscript revision.

### Conflict of Interest Statement

The authors declare that the research was conducted in the absence of any commercial or financial relationships that could be construed as a potential conflict of interest.
